# Unrevealed mosaicism in the next-generation sequencing era

**DOI:** 10.1007/s00438-015-1130-7

**Published:** 2015-10-19

**Authors:** Marzena Gajecka

**Affiliations:** Department of Genetics and Pharmaceutical Microbiology, Poznan University of Medical Sciences, Swiecickiego 4, 60-781 Poznan, Poland; Institute of Human Genetics, Polish Academy of Sciences, Strzeszynska 32, 60-479 Poznan, Poland

**Keywords:** Mosaicism, Mosaic ratio, Germline, Somatic, Constitutional, NGS

## Abstract

Mosaicism refers to the presence in an individual of normal and abnormal cells that are genotypically distinct and are derived from a single zygote. The incidence of mosaicism events in the human body is underestimated as the genotypes in the mosaic ratio, especially in the low-grade mosaicism, stay unrevealed. This review summarizes various research outcomes and diagnostic questions in relation to different types of mosaicism. The impact of both tested biological material and applied method on the mosaicism detection rate is especially highlighted. As next-generation sequencing technologies constitute a promising methodological solution in mosaicism detection in the coming years, revisions in current diagnostic protocols are necessary to increase the detection rate of the unrevealed mosaicism events. Since mosaicism identification is a complex process, numerous examples of multistep mosaicism investigations are presented and discussed.

## Introduction

**Fig. 1:**
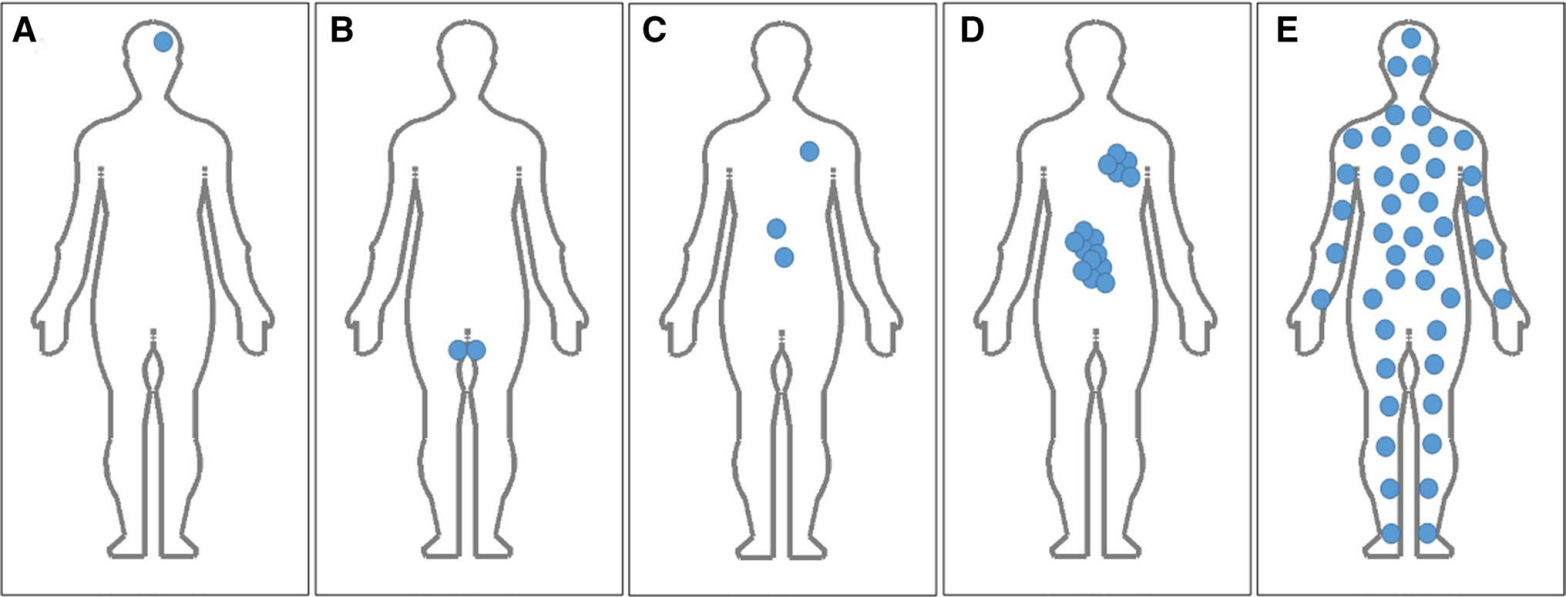
Various distribution of mutant cells in the human body and different types of mosaicism in particular individuals. In somatic mosaicism (**a**) mutant cells may appear with different mosaic ratio in patient’ distinct body tissues but not in gonads. As gonadal mosaicism refers to genetic variation in the genomes of germline cells within an individual it may be recognized in testes (**b**) and ovaries. Examples of low-grade (**c**) and medium-grade (**d**) somatic mosaicism in endoderm derivatives (epithelial lining of digestive tract and respiratory tract) point to quantitative difference in mosaic ratio. Constitutional somatic mosaicism (**e**) refers to the presence of normal and abnormal cells with a mutation recognized with the constant mosaic ratio (or almost unchanging mosaic ratio) in various tissues in the examined individual

Mosaicism in genetics is the presence in an individual of two or more cell lines that are genotypically distinct and are derived from a single zygote. Therefore, in mosaic body two or more distinct genotypes exist in different cell populations (Fig. 1). Germline (gonadal) mosaicism refers to genetic variation in the genomes of germline cells within an individual. In somatic mosaicism, in accordance with the newest research findings, mutation originated in somatic cell (somatic mutation) in early embryonic development may be found in both somatic and germline cells of this organism and may also lead to genotypic and possibly phenotypic heterogeneity within and between tissues (Holstege et al. 2014). Sporadic de novo mosaicism appears in the particular individual only. The parental mosaicism may manifest in the form of changes in the parent offspring’ phenotype as a result of both types somatic or germinal mutations. Current studies suggest that both sporadic and parental mosaicism may be more common than previously suspected (Donkervoort et al. 2015). Also, the apparently de novo mutation in the patient might be indeed a consequence of somatic mosaicism identified in somatic tissues of unaffected parents of this patient (Campbell et al. 2014a).

In general, in somatic mosaicism, different mosaic ratio may be found in patient’ distinct body tissues. Constitutional somatic mosaicism refers to the presence of normal and abnormal cells with a mutation recognized with the constant mosaic ratio in various tissues in the examined individual.

Mosaicism arises as a result of genetic alterations of different type and size, including single nucleotide variants (SNVs), copy number variants (CNVs), and simple or complex chromosomal rearrangements. These alterations may be present only in a subset of somatic or germ cells in the individual’ body. The issues when each mutation was acquired and how the analyzed clones expanded during a lifetime remain to be elucidated (Holstege et al. 2014). However, based on the recent research findings and clinical diagnostics data, it is suggested that the occurrence of mosaicism events is underestimated as the genotypes in the mosaic ratio often stay unrevealed, especially in the low-grade mosaicism.

## Somatic mutations are frequent events during early development and aging

Somatic mutations commonly occur during cell division; therefore, frequently dividing cells are more prone to acquire somatic mutations than tissues that rarely divide (Youssoufian and Pyeritz 2002). Epithelial cells, hematopoietic cells, and male germ cells are examples of repeatedly dividing cell types which are vulnerable to somatic mutations leading to phenotype alterations (Campbell et al. 2014a, b). In general, every round of mitotic cell division is expected to generate several somatic mutations (Youssoufian and Pyeritz 2002; Seshadri et al. 1987). It was assumed that the effects of somatic mutations were restricted to the lifetime of the individual and were not transmitted to the progeny (De 2011). The current research revealed that somatic mutation acquired in the early stage of embryonic postzygotic development might cause mosaicism in the parent who later transmits the mutation to the offspring through the germ cell (Campbell et al. 2014a).

Mosaicism has been reported in as high as 70 and 90 % of cleavage- and blastocyst-stage embryos derived from in vitro fertilization, respectively (Taylor et al. 2014). Postzygotic chromosome loss, chromosome gain and mitotic nondisjunction were observable in some cells creating mosaic events in early embryos (Daphnis et al. 2005; Taylor et al. 2014).

Very interested study model to assess somatic variation occurring during early embryonic development is this involving monozygotic twins. Among other findings, twins discordant for somatic mosaicism for aneuploidy of chromosomes X and Y (Razzaghian et al. 2010) and different copy number profiles changing during aging in monozygotic twins have been identified (Dumanski 2008). Also, it has been detected that deletions, inversions and translocations of genetic material were more common in aging mice compared with younger ones (Dollé and Vijg 2002) causing in aging tissues increasingly heterogeneous gene expression.

Also, mitochondria accumulate altered sequence variants during the lifetime. Mitochondrial genetics is different from the Mendelian in many aspects, including the uniparental inheritance of disease mutations and the presence of many copies of the genome within a single cell. Mitochondrial DNA (mtDNA) mutations are an important cause of inherited diseases. Mitochondrial diseases display pathological phenotypes because of the mixture of mutant versus wild-type mtDNA, known as heteroplasmy. Similarly to somatic DNA mutation, the mtDNA alteration may occur in a part of mitochondria only. Disease causing heteroplasmy can be identified at several levels, including within the multiple copies of mtDNA per mitochondria, between healthy and diseased mitochondria within a cell, or among mosaic cellular subpopulations assembled within tissues (Clifford et al. 2013).

Methods in the detection of the degree of heteroplasmy are similar to mosaicism identification in nuclear DNA. Increasing sensitivity of genomic technologies supports mitochondrial heteroplasmy assessment at the genome-wide scale (Li et al. 2010; Sosa et al. 2012; Li and Stoneking 2012). A number of studies using the newest approaches allowed detection of medium- and high-frequency heteroplasmy. It has been found that ~25–65 % of the general population had at least one heteroplasmy across the entire mitochondrial genome (Li et al. 2010; Sosa et al. 2012).

Therefore, in the optimal mosaicism search scenario, mosaicism occurrence in the cells in early development stage and in the terminally differentiated tissues, and in both nuclear DNA and mtDNA should be considered and investigated to characterize entirely mosaicism phenotypic consequences in the examined individual. Still, in laboratory practice usually one or two tests are performed, frequently chosen to be used based on incorrect assumptions as discussed later in this paper.

There are numerous endogenous molecular mechanisms that generate somatic mutations and cause mosaicism functioning during the lifetime of an individual, whereas others (such as Alu and L1 retrotransposition) are likely to have specific temporal patterns (De 2011). The mechanisms give rise to genetic sequence variants of predictable or unknown phenotypic consequences. DNA damage by reactive oxygen species, replication error by DNA polymerase and erroneous DNA repair, DNA polymerase slippage and trinucleotide repeat expansion, both short and long interspersed nuclear element (Alu and L1) retrotransposition, fork stalling and template switching (FoSTeS), non-homologous end joining (NHEJ), non-allelic homologous recombination (NAHR), micro-homology-mediated replication-dependent recombination (MMRDR), micro-homology-mediated break-induced repair (MMBIR), reversion mosaicism, and loss or gain of chromosomes of ploidy have been reported as involved in mosaicism etiology, as reviewed in detail in the paper by De (2011). Exogenous factors such as nicotine and alcohol usage, and UV exposure may also be involved in somatic mutation formation.

To emphasize the high incidence of somatic mutations, revertant mosaicism should be mentioned. Revertant mosaicism is a naturally occurring phenomenon relatively common in genetic skin diseases (Pasmooij et al. 2012). In revertant mosaicism, cells carrying disease-causing mutations co-exist with cells in which the inherited mutation is genetically corrected by a spontaneous event (Pasmooij et al. 2012). It has been found that in vivo reversion of somatic cells involved the skin, the liver and the hematopoietic cells, thus the tissues with high cell proliferation rates (Davis et al. 2010). Also, revertant mosaicism has been noticed repeatedly within the same patient and in vivo reversion could involve multiple cell lineages or be limited to a particular cell clone (Lai-Cheong et al. 2011).

All those data indicate that number of somatic mutations may be higher than anticipated in both healthy and changed by disease tissues. As a consequence, somatic mosaicism incidence would be presumably underestimated.

## Harmless nature of somatic mutations in healthy human tissues

Several reports revealed the increase in occurrence of somatic copy number changes with age in several tissues in mice and in peripheral blood in cancer-free humans as discussed in the paper by Holstege et al. (2014). Recently, biological material derived from a healthy person of extreme age, a 115-year-old woman, was examined to test the hypothesis that the number of single nucleotide somatic mutations might increase with age (Holstege et al. 2014). The examined woman had no symptoms of hematological illnesses, and autopsy showed that she did not suffer from vascular- or dementia-related pathology. She had breast tumor surgery at age 100 and died 15 years later of a gastric tumor that metastasized into her abdomen (Holstege et al. 2014). She has never received mutation inducing chemotherapy. DNA was isolated from several tissues that were collected during autopsy: whole blood, brain (occipital cortex), artery (media and endothelium), kidney (renal pyramid and minor calyx), heart, liver, lung, spleen, aorta, and the gastric tumor that she died of. DNA was also isolated from the breast tumor that was removed at age 100 (Holstege et al. 2014). The prevalence and types of single nucleotide and small insertion/deletion mutations that are somatic within the healthy blood genome were assessed. Since cells in occipital brain tissue rarely divide after birth (Spalding et al. 2005), it was expected that these cells did not acquire many somatic mutations, so that DNA isolated from occipital brain tissue could serve as a control sequence of the germline genome (Holstege et al. 2014). Consequently, DNA sequence extracted from peripheral blood was compared with DNA from the brain tissue. Based on deep whole-genome sequencing, approximately 450 somatic mutations in the nonrepetitive genome within the healthy blood cells were found (Holstege et al. 2014). Detected and confirmed somatic SNVs and indels were mostly novel (Holstege et al. 2014). None of the 376 somatic mutations that mapped to coding regions were predicted to have a deleterious effect on protein function by the SIFT and PolyPhen algorithms. Furthermore, none of the mutations were previously associated with clinical outcome. Therefore, as somatic mutations overlapped with functional elements similar to nonpathogenic dbSNP variants, but did not with disease-associated variants, the data supported their harmless nature (Holstege et al. 2014). However, there are also contradictory data indicating the damaging effect of somatic mutations, as discussed below.

## Impact of mosaicism on the epigenome

Somatic mutations can possibly affect the epigenetic patterns and levels of gene expression, and then the phenotypes of cells. Also, regardless of the sequence alterations in nuclear and/or mitochondrial DNA, abnormal DNA methylation, as an example of the so-called epi-mutations, is frequently observed (Holstege et al. 2014; Berko et al. 2014; Laurentino et al. 2015).

Likewise to genetic variation, changed methylation patterns may occur in a subset of cells only. In the body of 115-year-old woman, mutations occurred in cells with a stem cell-like methylation signature (Holstege et al. 2014). The authors of that study suggested that a subset of the somatic mutations have resulted from the spontaneous deamination of methylated cytosines, forming a thymine at that location. They found that 62 of the 376 somatic mutations mapped in putatively methylated CpG sites, indicating a significantly increased mutation-likelihood at CpG loci (*P* value <1 × 10^−6^) (Holstege et al. 2014).

In a paper by Berko et al., mosaic epigenetic dysregulation of ectodermal cells in autism spectrum disorder (ASD) was revealed (Berko et al. 2014). To explore whether advanced maternal age involves hidden aneuploidy or epigenetic dysregulation leading to ASD in the offspring, a homogeneous ectodermal cell type from 47 individuals with ASD compared with 48 typically developing controls born to mothers of ≥35 years, using a quantitative genome-wide DNA methylation assay, was tested (Berko et al. 2014). The results pointed to the presence of a mosaic subpopulation of epigenetically dysregulated, ectodermally derived cells in subjects with ASD (Berko et al. 2014). The results indicated that epigenetic dysregulatory mechanisms might complement and interact with DNA mutations in the pathogenesis of the disorder (Berko et al. 2014).

In the latest report, epigenetic germline mosaicism in infertile men was assessed (Laurentino et al. 2015). As abnormal sperm parameters and male infertility have been previously linked to aberrant methylation patterns of imprinted genes in sperm DNA, the authors of this study investigated whether spermatozoa were a homogeneous cell population regarding DNA methylation of imprinted genes (Laurentino et al. 2015). Pyrosequencing-based oligo-sperm methylation assay (OSMA) and deep bisulfite sequencing were used to measure DNA methylation of the *KCNQ1OT1*, *MEST*, *H19* and *MEG3* genes. The study revealed that normozoospermic samples had a homogenous pattern of DNA methylation, whereas oligoasthenozoospermic samples contained discrete populations of spermatozoa with either normal or abnormal methylation patterns, indicating epigenetic mosaicism (Laurentino et al. 2015).

Further research is necessary to assess interactions between somatic and epigenetic mutations and phenotypical consequences of those relations, especially if the mutations are present in the mosaic pattern.

## Mosaic chromosomal abnormalities

Chromosomal abnormalities include an altered number of chromosomes, simple or complex chromosomal rearrangements involving deletions, duplications, insertions, inversions and translocations, and small supernumerary marker chromosomes. Mosaicism is frequently associated with those chromosomal mutations. It has been found that the ratio of mosaic cells may influence the severity of phenotypic changes (Kaminker et al. 1985; Liehr et al. 2013).

An altered number of chromosomes most commonly arises because of an error in chromosome distribution at cell division. The gains or losses of entire chromosomes may occur as a result of nondisjunction, anaphase lagging of both an autosome and a sex chromosome (Coonen et al. 2004) and chromosome gain referred to as endoreplication (Taylor et al. 2014).

Examples of syndromes associated with mosaicism are Trisomy 8 syndrome and Trisomy 9 mosaic syndrome. Most of the patients with Trisomy 8 syndrome have been mosaics of trisomy C-group autosome/normal (Kurtyka et al. 1988). In many cases, normal karyotype from cultured leukocytes but trisomy 8 in skin fibroblast cells was observed. There appears to be a lack of correlation between the phenotype and the percentage of trisomic cells (Smith 2006). In contrast to Trisomy 8 syndrome, in Trisomy 9 mosaic syndrome the incidence and severity of malformations and intellectual deficiency correlate with the percentage of trisomic cells in the different tissues (Kaminker et al. 1985; Smith 2006).

In addition to errors in chromosome number, chromosomal imbalance can result in shorter loss or gain of genetic information. In Pallister–Killian mosaic syndrome, the chromosomal change responsible for the disorder typically occurs as a random event during the formation of reproductive cells in a parent of the affected individual. As an error in cell division called nondisjunction, the isochromosome 12p is created and the child may have two normal copies of chromosome 12 along with an isochromosome 12p. As cells divide during early development, some cells lose the isochromosome 12p, while other cells retain the abnormal chromosome, causing mosaicism. Almost all cases of Pallister–Killian mosaic syndrome are caused by mosaicism for an isochromosome 12p (Izumi and Krantz 2014).

Chromosomal deletions and/or duplications or triplications of different sizes are being identified. Large copy number alterations, in size up to ~10 Mb, often arise as a result of erroneous non-allelic homologous recombination and non-homologous end joining (Dittwald et al. 2013). Also, in somatic mosaicism’ etiology, a role of interspersed nuclear elements-1 (LINE-1 or L1) has been established with L1 retrotransposons active during embryonic development causing copy number alterations (van den Hurk et al. 2007).

One more example of chromosomal abnormality is somatic mosaicism in cases with small supernumerary marker chromosomes (sSMC) (Liehr et al. 2013). Somatic mosaicism was found in slightly more than 50 % of sSMC carriers, in different mosaic rates, which might go below 5 % of the studied cells (Liehr et al. 2013). Even though in the majority of the patients somatic sSMC mosaicism had no direct clinical effect, there were also cases with altered clinical outcomes due to mosaicism (Liehr et al. 2013).

Since the genotype–phenotype correlations are difficult to be established in patients with constitutional chromosomal rearrangements (Gajecka et al. 2007), different mosaic rates of chromosomal abnormalities may additionally complicate the prediction of clinical outcome. Both recurrent and nonrecurrent chromosomal aberrations usually cause a range of phenotypic features observed in the studied patient groups (Gajecka et al. 2007, 2008a). However, in the laboratory practice, cases with high-grade mosaicism, with mutation in 70–99 % of cells, are not separated from the completely constitutional, meaning observed in 100 % of body cells, aberrations. In the coming years, with improved mosaicism identification protocols the phenotypic differences among patients with apparently the same mosaic chromosomal aberration but diverse mosaic ratio would be probably explained.

## Unrevealed low-grade mosaicism

High-grade mosaicism, in rate of 70 % and higher, usually stays unrecognized and then is not evaluated in clinical practice. In contrast, medium- and low-grade mosaicism, if suspected, usually is carefully assessed. Types of the tested biological materials and applied identification and verification methods constitute main factors influencing the mosaicism detection, especially of the low grade, with mosaic rates below 5 % of the studied cells. Besides the extensive effort and continuous progress, identification of mosaics remains very challenging in clinical diagnostics and research laboratories.

Mosaic chromosomal abnormalities have been reported to represent 8 % of abnormal laboratory chromosomal microarray analysis (CMA) results, suggesting that mosaic findings may be more common than previously thought (Ballif et al. 2006). Low-level mosaicism may be missed by standard cytogenetic techniques such as G-banded karyotype. The abnormality may not be reported if the percentage of abnormal cells does not meet the laboratory’s threshold for reporting. When mosaicism is suspected, in addition to standard protocols, extra cells may be analyzed as well various tests performed including fluorescence in situ hybridization (FISH) analysis using specific probes. Even when mosaicism is strongly suspected by the clinician, the putative genetic cause is usually unknown (Cheung et al. 2007), causing difficulties in the further genetic investigation. Because of relatively low resolution of cytogenetic methods, the submicroscopic abnormalities may remain undetected. While the analyses are frequently performed on the cultured cells, the detection of mosaicism event can be even more complicated by a loss or reduction of the abnormal cell line during cell culture protocols carried in vitro in laboratory (Ballif et al. 2006; Theisen et al. 2009). Those aspects are crucial to be considered especially if low-grade mosaicism is expected. Application of CMA in diagnostics significantly improved mosaicism identification process in patients presenting with chromosomal abnormalities (Wapner et al. 2012). Still, proper design of the test with multiple control probes and repeats, its sufficient resolution and the appropriate biological material tested are critical to solve the diagnostic problem.

Mutations much smaller in size, including indels or SNVs, are not detectable using conventional cytogenetic or molecular cytogenetic techniques. As a solution, molecular biology methods, including various PCR techniques, Sanger sequencing, MLPA, array SNP, the next-generation sequencing (NGS) and others, are implemented to identify, categorize, confirm or corroborate mosaicism findings. Despite numerous techniques available to be applied in the search, low-grade mosaicism remains a difficult issue in both clinical diagnostics and research.

## Tested material influences the mosaicism detection rate

Various biological materials may be tested in mosaicism investigation. Primarily, low invasive procedures are recommended in the sample collection process. Peripheral blood samples (PBSs), lymphoblastoid cell lines, amnion or chorion cells, fibroblasts, myocytes and cells derived from other tissue biopsies, buccal tissue, saliva, and nails are the most frequently used.

Mosaicism detection is always correlated with the mutation identification per se. Targeted material, containing the causing mutation, would be optimal for testing and always better than the most accessible one. As already mentioned, frequently dividing cells would be the examples of material to test or verify somatic mosaicism. In research, rarely dividing cells after birth, including occipital brain tissue, would constitute a control material in comparison analyses (Spalding et al. 2005; Holstege et al. 2014). In contrast, in constitutional somatic mosaicism the most accessible tissue sample will be appropriate for testing.

Germinal mosaicism is difficult to assess, especially if maternally originated mutation is suspected as the causative alteration, as eggs from ovaries are difficult to be derived, besides the aspect of highly invasive procedure.

Usually the type of material is chosen based on diagnostic technique to be applied. Of the almost 400 different human body tissues, generally only one tissue, i.e., blood, amnion or chorion cells, or fibroblasts is studied cytogenetically (Liehr et al. 2013). This fact generates a risk of inaccuracy in mosaicism assessment. Based on the chosen material, a simple sample or even multiple samples, the real rate of somatic mosaicism in any studied individual is impossible to be precisely recognized (Liehr et al. 2013). Since somatic mutations at the same time may occur in different tissues and in distinct body’ sites with different mosaic ratio (Fickelscher et al. 2007; Liehr et al. 2011), without extensive sampling the obtained results would be correct for the specific analyzed samples only. For example, deep resequencing of a mutation on a next-generation sequencer identified very-low-grade somatic mosaicism in the mother: 0.4, 1.1, and 8.3 % in the saliva, blood leukocytes, and nails, respectively (Miyatake et al. 2014) indicating various mosaic rates in the different tested tissues. This is why in the present-day genetic counseling the fact of mosaicism rather than mosaic ratio is further characterized. Still, the mosaic ratio is estimated to collect data for further genotype–phenotype correlations. In contrast to the latter research example, in a patient with breast cancer, very similar level of mutation (~5 %) in the tested tissues (in leukocytes, buccal tissue and normal breast tissue DNA) was identified and the authors concluded that the patient had a low-grade constitutional somatic mosaicism resulted in ~47 % mutation level in tumor tissue (Friedman et al. 2015).

At this point, it is worth to add that there is a growing concern in the field about whether some CNVs categorized as germline-acquired mutations in biobanks are actually somatically acquired alterations, especially when the germline mutations were inferred by testing only one tissue as pointed by De (2011).

The newest evidence has shown that acquired somatic mutations might remain specific during aging in the original sites of appearance only. In the 115-year-old woman, somatic mutations detected in blood were not detected in the breast cancer that patient had at age 100 nor in the gastric tumor she had at age 115 or other native tissues. This indicates that the somatic mutations in blood were not derived from tumor cells at the time of her death (Holstege et al. 2014). In that study, validation panel was also used to test samples derived from aorta, artery (endothelium), heart, and kidney (renal pyramid) tissues derived from 115-year-old woman’ body (Holstege et al. 2014). None of the confirmed somatic mutations detected in blood were detected in these tissues, and only an occasional mutation detected in blood could be detected in artery (media), kidney (minor calyx), liver, and spleen tissues (Holstege et al. 2014). On the other side, in accordance with current knowledge (Schwarzenbach et al. 2011), mutations identified in the tumor samples would be probably detectable in blood samples by the assessment of the cell-free circulating DNA. However, this analysis has not been performed.

Contamination is always the issue in daily laboratory practice. Again in the study of the 115-year-old woman, almost all identified somatic mutations were detected in DNA derived from the lung tissue (Holstege et al. 2014). The authors of that study suggested that the DNA isolated from lung tissue was apparently contaminated with blood DNA due to a vast leukocyte presence in the lung tissue (Holstege et al. 2014). One more example of sample contamination would be buccal tissue contaminated with saliva obtained during the oral swab procedure. In contrast to common opinion that buccal epithelial cells are mostly found in saliva, it has been reported that up to 74 % of the DNA in saliva comes from white blood cells (Thiede et al. 2000).

In the era of constant progress in technology used in clinical diagnostics and research, it is crucial to adjust the tested material to the detection technique. Sdano et al. reported on three patients in which CMA technology detected mosaicism with a larger percentage of abnormal cells in buccal tissue compared to blood leukocytes (Sdano et al. 2014). The authors recommended to clinicians to consider buccal samples as a primary sample for certain genetic studies (such as CMA) or to consider additional testing using DNA from buccal samples in patients for whom a genetic etiology was suspected but a diagnosis has not been achieved using DNA from blood leukocytes (Sdano et al. 2014). Blood leukocytes have long been the “gold standard” material for cytogenetic and other analyses. While the DNA derived from PBSs was usually tested in CMA approaches, the mentioned study and other current reports (Miyatake et al. 2014; Braunholz et al. 2015) evidently question the recommendations of standard diagnostic protocols.

Lymphoblastoid cell lines are controversial diagnostic material, very valuable to repeat the original experiments especially if the derived biological sample was used in the analyses but causing difficulties in result interpretation if unforeseen events took place during the cell line establishment process. Lymphoblastoid cell lines are commonly used in research laboratories. However, they frequently undergo both the introduction of large mosaic abnormalities and the loss of biological mosaicism due to a tendency toward clonality (Shirley et al. 2012; Migeon et al. 1988). The culturing process for routine chromosome analysis can complicate the detection of mosaicism since the normal cell line may have a growth advantage in the culture (Sdano et al. 2014). Even ‘simple’ cultured cells, proceeded without a step of immortalization, may be significantly altered and generate erroneous results in cytogenetic testing (Ballif et al. 2006; Theisen et al. 2009). Moreover, the immortalization process may cause not only mosaic ratio fluctuations but even additional chromosomal abnormalities, including terminal deletions (Gajecka and Shaffer 2007, unpublished data). It has been repeatedly confirmed, using FISH and sequence specific PCR techniques, that immortalization process generated terminal deletion of 1q44 chromosomal region in patients with 1p36 microdeletion syndrome, significantly affecting research outcomes when FISH probes corresponding to 1q44 region were used as control probes in the 1p36 microdeletion syndrome study (Gajecka and Shaffer 2007, unpublished data; Gajecka et al. 2006a).

A choice of appropriate tissue from a variety of biological materials to be tested seems to be even more difficult if numerous diverse techniques are available to be applied in the mosaicism detection, as discussed below.

## Applied methods impact the mosaicism identification

Chromosomal mosaicism is identified using various cytogenetics techniques, such as trypsin treatment and Giemsa staining, and synchronized culture techniques, to allow for the identification of individual chromosomes. Molecular cytogenetic techniques, like metaphase, interphase and fiber FISH and comparative genomic hybridization (CGH) as well as array comparative genomic hybridization (aCGH), allow for the detection of more subtle changes in copy number in the genome. In addition, various PCR techniques and sequencing of chromosomal regions containing the rearrangement breakpoints and junctions may be useful for characterization of specific rearrangements (Gajecka et al. 2006a, b, 2008b; D’Angelo et al. 2009; Rosenfeld et al. 2011; Midro et al. 2014). Depending on the probe and the clinical question, these techniques offer advantages in clinical situations including expected mosaicism.

Next-generation sequencing has revolutionized the field of genetics and currently provides opportunities to assess mosaicism, including the low-grade mosaicism. The high‐throughput nature of NGS technology allows for very high fold coverage of sequenced fragments and detection of low levels of mutated variants among wild-type alleles. Through the high sensitivity of this method, researchers are able to discover mosaic mutations often regarded as background noise and missed in Sanger sequencing (Rohlin et al. 2009). Recently, the targeted high sensitive NGS technique allowed for the detection of mosaic mutations present in only a small fraction of the blood cells in patients with brain malformations (Jamuar et al. 2014). The 63 % of these mosaic mutations were undetectable with the direct Sanger sequencing method but were validated through subcloning and subsequent sequencing of the subcloned DNA (Jamuar et al. 2014). NGS method was also used in the single-cell whole-genome sequencing in more than 200 single cells, including ~160 neurons from non-pathological and pathological human brains (Cai et al. 2014). In that study, mosaic clonal CNVs were identified in normal lymphoblast cells and neurons. These results are largely in agreement with a previously reported study (McConnell et al. 2013) and showed that NGS technologies were suitable for identification of mosaic CNVs. Taken together, NGS is an important diagnostic tool for detecting somatic mosaicism. However, a relatively high cost of an experiment may be a major limitation for large-scale application of this method in diagnostic laboratory.

Discussed below are different mosaicism investigations with various outcomes. The six examples, summarized in Table 1, were intended to show the complexity of mosaicism detection and complementarity of different methods, including NGS and other techniques, in the mosaicism study.

**Table 1:** Examples of mosaicism identification process in clinical diagnostics and research

	Inquiry/disease/phenotype No. of individuals tested	Material tested^a^	Method applied	Results	Remarks	References
1.	Choice of material and limitations of Sanger sequencing in molecular diagnostics/Cornelia de Lange syndrome/three unrelated patients (A, B and C)	(a) PBS and BM	(a) Sanger sequencing	(a) No disease-causing mutation in any of the five known CdLS genes	Mosaicism to be considered in CdLS Because of the limited availability of fibroblast cells for most of the patients there is an urgent need of modern and sensitive sequencing technologies in routine molecular diagnostics for CdLS	Braunholz et al. (2015)
		(b) PBS (patient B only)	(b) Exome sequencing	(b) No disease-causing mutation		
		(c) PBS (patients B and C only)	(c) CGH array	(c) No disease-causing mutation		
		(d) BM	(d) NGS, high-coverage^b^	(d) Three mosaic *NIPBL* mutations		
		(e) PBS	(e) SNaPshot assays	(e) No signal indicating the mutant allele was detected in patients A and B, only a very faint signal detected in patient C		
		(f) BM, fibroblast samples; urine (patients A and C only)	(f) SNaPshot assays	(f) Three mosaic *NIPBL* mutations		
		(g) Fibroblast samples	(g) Sanger sequencing	(g) Three mosaic *NIPBL* mutations		
2.	Somatic mosaicism in the healthy mother of affected siblings; novel heterozygous mutation in *ACTA1*/nemaline myopathy/two affected siblings, their both parents, and normal controls	(a) PBS	(a) Sanger sequencing	(a) Mutation in affected siblings only	Possibility of very-low-grade somatic mosaicism in suspected carriers, rather somatic than germline mosaicism HRM could not detect the 8.3 % mosaicism; NGS as the first choice for detecting very-low-grade somatic mosaicism	Miyatake et al. (2014)
		(b) PBS (siblings and parents)	(b) NGS, whole-exome sequencing (Illumina HiSeq2000)	(b) Mutation in affected siblings only		
		(c) Saliva, hair, nails, PBS	(c) Sanger sequencing	(c) No mutation in parents		
		(d) PBS (siblings), and saliva, nails, hair and PBS (parents)	(d) NGS, deep targeted resequencing for the mutation in *ACTA1* (Illumina MiSeq)	(d) Somatic mosaicism in the mother: 0.4, 1.1, and 8.3 % in saliva, PBS, and nails, respectively		
		(e) PBS (siblings and parents), and saliva, nails, and hair (mother only)	(e) Allele-specific PCR and Sanger sequencing of these amplicons	(e) Mutation in PBS of the siblings and the mother, and in saliva and nails of the mother		
		(f) PBS (normal controls, siblings, father), and nails (mother)	(f) HRM analysis	(f) The melting curves of both affected siblings were aberrant (mutant), those of the parents were called normal		
3.	Comparison of HRM analysis, pyrosequencing, allele-specific PCR, NGS and IHC to Sanger sequencing; detection of p.V600E and non-p.V600E *BRAF* mutations/melanoma/49–82 tumor samples per technique	(a–f) DNA extracted from formalin-fixed paraffin-embedded tissues: 63 samples were melanomas, 11 were lung adenocarcinomas and eight were colorectal carcinomas	(a) HRM analysis	(a) Time and cost saving, 100 % specificity, detection limit of 6.3 % mutated alleles	High sensitivity and multiplexing options of NGS allowing to generate a molecular profile of each tumor sample analyzed A combination of HRM and IHC recommended to increase sensitivity and specificity for routine diagnostic of *BRAF* mutations	Ihle et al. (2014)
			(b) Pyrosequencing^c^	(b) 90 % specificity, detection limit of 5 % mutated alleles, result interpretation prone to errors		
			(c) Allele-specific PCR^d^	(c) Limited utilization detecting p.V600E mutations only with 98.3 % specificity and detection limit of 7 % mutated alleles		
			(d) Targeted NGS (Illumina MiSeq)	(d) Should be carefully validated before implementation into routine diagnostics, 100 % specificity, detection limit of 2 % mutated alleles		
			(e) IHC^e^	(e) Fast and cheap performance, 98 % specificity, 100 % sensitivity, detection limit of 5 % mutated alleles		
			(f) Sanger sequencing	(f) A reliable method, detection 100 % specificity, detection limit of 6.6 % mutated alleles		
4.	Clinical utility of CMA using DNA from buccal cells/ASD and other phenotypic features (patient 1), developmental delay and ASD (patient 2), hypergonadotrop hichypogonadis, short stature and mild learning disabilities (patient 3)/three unrelated patients	(a) BC (patient 1 only)	(a) CMA, with both copy number and SNP probes^f^	(a) A gain of chromosome 21, consistent with 15–20 % mosaicism for trisomy 21	Buccal samples as a primary sample for certain genetic studies (such as CMA) Additional testing using DNA from buccal samples to be considered in patients for whom a genetic etiology is suspected but a diagnosis has not been achieved using DNA from blood leukocytes	Sdano et al. (2014)
		(b) CL (patient 1 only)	(b) FISH (500 interphase nuclei, four probes)	(b) 22/500 interphase nuclei with extra copy of chromosome 21		
		(c) CL (patient 1 only)	(c) G-banded chromosome analysis (100 metaphase cells)	(c) 4/100 metaphases with extra copy of chromosome 21		
		(d) PBS (patient 2 only)	(d) CMA^g^	(d) No abnormalities		
		(e) BC (patient 2 only)	(e) CMA, with both copy number and SNP probes^f^	(e) 20 % mosaicism for tetrasomy 12p, diagnosis of Pallister–Killian syndrome		
		(f) CL (patient 3 only)	(f) Standard G-banded karyotype ( 20 metaphase cells)	(f) No abnormalities		
		(g) CL (patient 3 only)	(g) Standard G-banded karyotype (46 more metaphase cells)	(g) Four of the 66 total cells were found to be 45,X		
		(h) BC (patient 3 only)	(h) CMA, with both copy number and SNP probes^f^	(h) Three cell lines suspected: 45,X (in ~45 % of BS and 6 % of CL), 46,XX, and the indeterminate cell line [either (46,X,idic (X)(p11.21) or 46,X,del (X) (p11.21)] in ~15–20 % of BS and not seen in CL Normal chromosome complement in ~40 % of BS and 94 % of CL		
5.	Quantitative analysis of the ratio of mutant versus wild-type allele (*COL6A1*, *COL6A2*, and *COL6A3*) in genomic DNA from various tissues/COL6-RD/four families	(a) PBS, CDF, and saliva (family 1 only)	(a) Real-time PCR	(a) Too weak signal to be detected in PBS and saliva from the mosaic father, 36 % of the mutant allele in the CDF the mosaic father	Somatic mosaicism for dominant collagen 6 mutations suggests that parental mosaicism may be more common than previously suspected in COL6-RD	Donkervoort et al. (2015)
		(b) PBS, CDF, saliva, and cDNA from CDF (family 2 only)	(b) Real-time PCR	(b) 20, 42, and 16 % of mutant allele ratio in the father, in PBS, CDF and saliva, respectively, while 35 % mutant allele ratio from the cDNA from CDF		
		(c) CDF (family 3 only)	(c) Real-time PCR	(c) 24 % of mutant ratio in CDF of the mosaic father		
		(d) PBS and saliva (family 4 only)	(d) Real-time PCR	(d) 40 % of mutant ratio in PBS and 58 % in the saliva of the mosaic mother		
		(e) Fibroblasts (patients and parents, families 1, 2, and 3 only), PBS (patients and parents, family 4)	(e) Sanger sequencing	(e) Heterozygous mutant alleles identified in the four patients and in one of their parents; peak height of the mutant allele in the carrier parents was significantly smaller		
6.	Low-level constitutional mosaicism of a de novo *BRCA1* c.1953dupG gene mutation/breast cancer/one case	(a) PBS (blood draw 1)	(a) NGS (Illumina MiSeq, HiSeq 2500) of 29 hereditary cancer genes^h^	(a) Pathogenic *BRCA1* mutation in 5 % of reads	NGS sequencing should be considered in affected individuals whose tumors display a *BRCA* mutation that cannot be demonstrated in PBS using Sanger sequencing	Friedman et al. (2015)
		(b) PBS	(b) Sanger sequencing of *BRCA1* and *BRCA2*	(b) No mutation		
		(c) PBS	(c) Sanger sequencing of c.1953dupG mutation locus	(c) The presence of a small peak of c.1953dupG mutation, with signal almost indistinguishable from background noise		
		(d) PBS (both blood draw 1 again and blood draw 2) and buccal swab	(d) NGS (Illumina MiSeq, HiSeq 2500) of 29 hereditary cancer genes^h^	(d) Mosaic signal of 4.9–6.8 % was reproducibly detected in all samples		
		(e) Breast tumor tissue	(e) Somatic NGS assay^i^	(e) Mutation c.1943dupG in 47 % of sequence reads		
		(f) Healthy breast tissue	(f) Somatic NGS assay^i^	(f) Mutation c.1943dupG was in 5 % of reads		
		(g) Maternal PBS	(g) Deep sequencing	(g) No c.1943dupG mutation		

^a^ *BM* buccal mucosa, *PBS* peripheral blood sample (lymphocyte DNA), *BC* buccal cells, *CL* cultured lymphocytes, *CDF* cultured dermal fibroblasts, *BT* buccal tissue

^b^Ion Torrent PGM with the AmpliSeq Designer Tool

^c^ *therascreen* ^®^ BRAF Pyro Kit (Qiagen)

^d^The cobas^®^ *BRAF* V600 test (Roche)

^e^Monoclonal mouse antibody VE1 (Spring Bioscience)

^f^Affymetrix CytoScanHD^®^, Affymetrix

^g^Affymetrix 6.0 array^®^

^h^NGS of 29 hereditary cancer genes, performed at Invitae (San Francisco, CA, USA)

^i^Reported as commercially available somatic NGS assay

In addition to NGS technologies, other methods are implemented in mosaicism identification including Sanger sequencing, high-resolution melting (HRM) analysis, allele-specific PCR, pyrosequencing, SNaPshot and immunohistochemistry. In the NGS era, those techniques would be especially useful in preliminary screening and result verification.

Sanger sequencing has been frequently used in mosaicism investigations. Limitations of Sanger sequencing in molecular diagnostics were currently shown in a study on Cornelia de Lange syndrome (CdLS) (Braunholz et al. 2015). The genetic cause of CdLS is a mutation in one of the five associated genes (*NIPBL*, *SMC1A*, *SMC3*, *RAD21*, and *HDAC8*) accounting for about 70 % of cases and the genetic cause of the remaining 30 % of the patients with a clinical diagnosis of CdLS is unknown (Braunholz et al. 2015). Braunholz et al. collected buccal mucosa (BM) samples of patients that were negative for mutations in the known CdLS genes testing DNA derived from the blood. Three mosaic *NIPBL* mutations by high-coverage gene panel sequencing approach that was undetected by classical Sanger sequencing of BM DNA were identified (Braunholz et al. 2015). All mutations were confirmed with a primer extension-based method developed for the analysis of SNPs (SNaPshot fragment analysis) using DNA from BM, urine, and fibroblast samples. The respective mutations were not detected in PBSs. Finally, in fibroblast samples from all three patients, Sanger sequencing could identify all the mutations (Braunholz et al. 2015) as summarized in Table 1 (*Example 1*).

At present, Miyatake et al. explored whether low-grade somatic mosaicism was detectable by HRM analysis (Miyatake et al. 2014). Low-grade somatic mosaicism in a suspected carrier, rather than germline mosaicism, has been revealed using various techniques (Miyatake et al. 2014), as shown in Table 1 (*Example 2*). The HRM has been suggested to be one of the more sensitive methods in mosaic mutation identification (Ihle et al. 2014). HRM analysis was performed using DNA from unaffected individuals (controls), the affected siblings, the father (all DNA derived from blood), and the mother [DNA derived from the nails, which showed the highest rate of mosaicism (8.3 %) by the NGS, targeted resequencing]. The melting curves of the two affected siblings were aberrant and were called mutant, while those of the parents were called normal. Therefore, HRM analysis could not detect the 8.3 % mosaicism (Miyatake et al. 2014). In contrast, allele-specific PCR was sufficiently sensitive to detect mosaicism in PBS derived from the mother in mosaic rate of 1.1 % (Miyatake et al. 2014). The authors of this study concluded that using the conventional mosaicism approach, the mother might have been judged to have germline mosaicism and, therefore, NGS should be the first choice for detecting very-low-grade somatic mosaicism (Miyatake et al. 2014).

In other current report, sensitivity, specificity and feasibility of six different methods for the detection of *BRAF* mutations (p.V600E and non-p.V600E) were evaluated. Comparison of HRM analysis, pyrosequencing, allele-specific PCR, targeted NGS and immunohistochemistry (IHC) to conventional Sanger sequencing was performed (Ihle et al. 2014) as presented in Table 1 (*Example 3*). Targeted NGS was shown to be able to detect somatic *BRAF* mutations down to 2 % allele frequency, demonstrating the increased sensitivity of this method compared with HRM (limit 6.6 % allele frequency), pyrosequencing (limit 5 % allele frequency), and Sanger sequencing (limit 6.6 % allele frequency) (Ihle et al. 2014). However, it is necessary to mention that assessed mutations were known as causative in melanoma and were previously extensively evaluated. Assessment of unknown mosaicism event without specific mutation indication in clinical diagnostics is definitely much more challenging.

As buccal cells are becoming increasingly utilized for clinical analyses and are proving to have many advantages, clinical utility of CMA analysis using DNA derived from buccal cells in free patients was investigated (Sdano et al. 2014), as shown in Table 1 (*Example 4*). *Patient 1* was determined to be mosaic for trisomy 21 with the abnormality present in 15–20 % of the buccal sample (BS) (using MCA) and 4 % of the cultured blood leukocyte (CL) sample (using interphase and metaphase FISH) (Sdano et al. 2014). In *patient 2*, the second CMA experiment, performed on a BS, showed an estimated 20 % mosaicism for tetrasomy 12p, consistent with a diagnosis of Pallister–Killian syndrome. Retrospective analysis of the results of the first CMA experiment (DNA from PBS used) showed no evidence of a copy number gain of 12p. Therefore, the latest finding was only detectable through analysis of the buccal specimen (Sdano et al. 2014). In *patient 3*, standard G-banded karyotype analysis (with increased number of analyzed cells) and CMA revealed three cell lines 45,X (in ~45 % of BS and 6 % of CL), 46,XX, and the indeterminate cell line [either (46,X,idic (X) (p11.21) or 46,X,del (X) (p11.21)] in ~15–20 % of BS and not seen in CL while the normal chromosome complement was present in ~40 % of BS and 94 % of CL (Sdano et al. 2014). These cases confirm that CMA analysis can detect mosaicism and that uncultured buccal samples may be the most suitable sample for at least the first line of genetic analysis, especially in the case of mosaicism suspicion (Sdano et al. 2014). Those three cases indicate the need to properly choose both the optimal biological material to be tested and one or more appropriate techniques in the mosaicism search. Applied CMA platform (CytoScanHD^®^, Affymetrix) included probes for single nucleotide polymorphic (SNP) identification and copy number responsive probes allowing for both single nucleotide variants and copy number changes identification, increasing confidence in detection of mosaicism.

In mosaicism identification, numerous PCR techniques are implemented, including real-time PCR, long-range PCR, and individual-specific breakpoint PCR. Quantitative analysis of the ratio of mutant versus wild-type allele (*COL6A1*, *COL6A2*, and *COL6A3*) in genomic DNA from various tissues, including blood, cultured dermal fibroblasts, and saliva, was performed in four families with collagen type 6-related dystrophies and myopathies (COL6-RD) (Donkervoort et al. 2015). Applied real-time PCR revealed differences between mosaicism ratio in tissues tested in parents carrying the causative mutations (Donkervoort et al. 2015), as presented in Table 1 (*Example 5*). Consistent with somatic mosaicism, parental samples had lower ratios of mutant versus wild-type allele compared with the fully heterozygote offspring (Donkervoort et al. 2015).

Pathogenic *BRCA1* mutations are usually inherited. Constitutional low-level *BRCA1* mosaicism has never been reported until the current paper published by Friedman et al. (2015). Using NGS of a cancer gene panel of germline and tumor DNA in a patient with early onset of breast cancer, constitutional de novo mosaicism (~5 %) for a pathogenic (c.1953dupG; p.Lys652Glufs*21) *BRCA1* mutation was detected in leukocytes, buccal tissue and normal breast tissue DNA, with ~47 % mosaic ratio of the mutation in tumorous breast tissue (Friedman et al. 2015), as indicated in Table 1 (*Example 6*). In previous reports of somatic *BRCA1* mutations, the mutant allele was identified in the tumor cells only, as pointed in the paper by Friedman et al. (Friedman et al. 2015). Although a few cases of de novo constitutional *BRCA1* or *BRCA2* mutations have previously been described, most were detected in a heterozygous form in constitutional DNA and were not mosaic (Friedman et al. 2015). In the study by Friedman et al., mosaic signal of 4.9–6.8 % was reproducibly detected in all samples of leukocytes, buccal tissue and normal breast. Therefore, the consistency of the load of c.1953dupG mutation across different tissue types suggested that this event occurred early in embryonic development and this idea was further supported by the lack of the same mutation in maternal constitutional DNA confirmed using deep sequencing (Friedman et al. 2015).

Campbell et al. assessed somatic mosaicism for transmitted mutations among parents of children with the simplex genetic disease (Campbell et al. 2014a). Individual-specific breakpoint PCR was applied to assess 100 families with children previously found to be affected by genomic disorders due to rare deletion CNVs originally determined to be de novo by clinical analysis of parental DNA (Campbell et al. 2014a). Performing highly sensitive individual-specific breakpoint PCR, they identified four cases of low-grade somatic mosaicism for the transmitted CNVs in DNA isolated from PBS (Campbell et al. 2014a). The authors concluded that the technique of individual-specific breakpoint PCR was more sensitive, less expensive, required less infrastructure and was less invasive than a skin biopsy in the CNV mosaicism detection (Campbell et al. 2014a). However, to design the final PCR the individual-specific breakpoint should be identified earlier in the affected individual to test the parents.

To detect large rearrangements, multiplex ligation-depending probe amplification (MLPA) is often used. It has been shown that MLPA was less sensitive in detecting low-grade somatic mosaicism than FISH or a mutation-specific PCR test (van Veghel-Plandsoen et al. 2011). MLPA of *TSC2* gene showed a pattern consistent with a duplication of sequence fragment containing exons 15–26. Using MLPA protocol, the duplication was hardly detectable in father’ DNA (van Veghel-Plandsoen et al. 2011). Long-range PCR, followed by sequencing of the abnormal PCR product, resulted in the identification of the mutation and the ratio of the mutant versus the wild-type fragment was lower compared with the proband, indicating that the father was a somatic mosaic for the mutation (van Veghel-Plandsoen et al. 2011). The duplication-specific PCR showed that about 30 % of the father’s leukocytes carried the duplication and this technique was able to detect a mosaic pattern of about 10 % (van Veghel-Plandsoen et al. 2011). A mosaic pattern of about 40 % could be detected by MLPA analysis, which explains why the 30 % mosaicism could not be detected by MLPA. To investigate the sensitivity of the MLPA in detecting deletions, material derived from patients with a total *NF1* deletion, previously identified using FISH, was assessed. It was possible to detect a deletion present in 22–30 % of the cells. These results indicate that mosaic duplications are harder to be detected than deletions using MLPA method (van Veghel-Plandsoen et al. 2011). Still, the size of the rearrangement, the probe density, and DNA quality could influence the mosaic mutation detection (van Veghel-Plandsoen et al. 2011).

Array-based comparative genomic hybridization (aCGH), as a one type of the CMA, has been frequently used in chromosomal abnormality investigations, especially if other routine techniques including subtelomere FISH did not reveal aberration. In a patient with a small (1.52 Mb), interstitial deletion in 1p36 region de novo rearrangement was identified using bacterial artificial chromosomes and oligonucleotide microarrays in the patient. Metaphase FISH with probes corresponding to the deletion region was performed and excluded the deletion in the parents. However, when a second child was born in this family performed CMA showed apparently the same interstitial deletion (Gajecka et al. 2010). As a germline mosaicism was suspected in the mother, the deletion breakpoints and the sequence junction in the rearrangement were identified using molecular biology techniques in the older sibling. Next, using breakpoint specific PCR with primers unique for the two interstitial deletion breakpoints, the PCR product containing the junction sequence fragment was amplified using DNA from blood samples derived from both siblings. That result confirmed that the same rearrangement occurred in both siblings and that the germline mosaicism in the mother was the possible pattern of the deletion occurrence (Gajecka et al. 2010). Currently, with the newest evidence concerning low-grade mosaicism in the healthy parents of the affected offspring, this research example would be a subject to suspect a low-grade mosaicism in the mother. However, based on the breakpoint-specific PCR performed with blood DNA samples of both parents, no amplification of the junction fragment was obtained, again suggesting germline rather than somatic mosaicism (Gajecka and Shaffer 2014, unpublished data).

Currently, microarray analyses, also known as molecular karyotyping (or CMA), are widely used in clinical diagnostics. Wapner et al. evaluated the accuracy, efficacy, and incremental yield of CMA as compared with karyotyping for routine prenatal diagnosis (Wapner et al. 2012). It was shown that microarray analysis was equivalent to a standard karyotype analysis for the prenatal diagnosis of common aneuploidies. The used array was designed to maximize the detection of well-characterized microdeletions and duplications but also included oligonucleotides to identify additional chromosomal imbalances (Wapner et al. 2012). Preferentially uncultured samples were proceeded to avoid, among others, the artifacts of cell and tissue culture (Wapner et al. 2012). It was noted that in 4391 samples available for microarray analysis 58 had mosaic karyotype results and those mosaic cases were excluded from the CMA. Microarray analysis identified all the aneuploidies detected by means of standard karyotyping. In addition, eight of these cases, all from uncultured chorionic-villus samples, were mosaic on the microarray and could represent mosaicism not detected on karyotyping (Wapner et al. 2012). Also, of the three marker chromosomes detected on karyotyping, two were identified on microarray (Wapner et al. 2012).

Single-cell array-based comparative genomic hybridization (single-cell aCGH) is an example of successful development of high-resolution full-genome analysis methods applicable to single cells. For example, Jacobs et al. studied the genetic content of 92 individual human cells, including fibroblasts, amniocytes and embryonic stem cells (hESCs), using single-cell array-based comparative genomic hybridization (aCGH) (Jacobs et al. 2014). They found that human somatic and embryonic stem cell cultures show significant fractions of cells carrying unique megabase-scale chromosomal abnormalities, establishing genetic mosaics that could not have been detected by conventional cytogenetic methods (Jacobs et al. 2014).

Also SNP arrays are utilized in mosaicism investigations, including patients with pregnancies at increased risk for the common aneuploidies (Van Opstal et al. 2015). SNP array allows for detection of submicroscopic chromosome abnormalities, presented also in the form of low-level mosaicism (Van Opstal et al. 2015).

High-resolution SNP microarray generates a huge number of data. This fact remains an issue in the proper diagnostics process. Since enhanced statistical analyses may probably resolve the problem, various methods are implemented in bioinformatics pipelines. The parent-of-origin-based detection (POD) method for chromosomal abnormality detection in trio-based SNP microarray data is an example of the solution showing the robustness across multiple types of Illumina microarray chips (Baugher et al. 2013). triPOD is a fast, efficient, multi-threaded software program for chromosomal abnormality detection in offspring using SNP array data from parent–child trios. The implementation of the POD method greatly increases the ability to detect mosaic abnormalities in SNP array data (Baugher et al. 2013). Although triPOD was developed for microarray data from the Illumina platform, automated adjustments for sample-specific levels of quality and variation allow for application to other platforms from which SNP-specific genotypes, allelic ratios, and copy number data can be derived (Baugher et al. 2013). Also, an adaptation of the POD method for analysis of mosaicism in next-generation sequence data is anticipated (Baugher et al. 2013).

Sometimes mosaicism is investigated with other than discussed above techniques. Suspicion of revertant mosaicism in the clinic can be investigated through skin biopsy analysis, typically using immunohistochemistry and/or transmission electron microscopy (Lai-Cheong et al. 2011). Also, real-time PCR and flow cytometry were applied in the mosaicism detection in patients with autosomal dominant hyper-IgE syndrome (Hsu et al. 2013).

## How multistep is mosaicism detection process?

Inconsistencies between mosaicism detection techniques and variety in the downstream analyses may complicate the comparison of the clinical diagnostic’ or research outcomes. Mosaicism identification is usually a multistep process, extensive, expensive and time consuming. Table 1 presents mosaicism detection process in different cases suspected to be mosaic. Usually more than one technique is used to recognize mosaicism. Then, additional method, usually different than the first one used, is applied to confirm the feature. In the case of performed qualitative assessment only the subsequent test will be done to estimate the mosaic ratio. Finally, with the clinical and diagnostic data, genotype–phenotype correlation will be assessed to discuss and/or predict mosaicism clinical consequences.

There is no gold standard test in mosaicism detection. While the improvement in mosaicism detection points to NGS technologies as the most promising method, there are some hesitations and exemptions. For example, it is postulated that the only reliable approach to detect sSMC present in the low-level mosaic pattern is banding cytogenetics (Liehr et al. 2013). Also, in the case of *Patient 3* (Sdano et al. 2014; Table 1, *Example 4*), the complexity of mosaicism events was finally revealed using CMA, with both copy number and SNP probes. However, only trained in G-banded karyotype analysis cytogeneticist could verify the finding (Sdano et al. 2014). Moreover, it is worth to point that methods already established and functioning in the laboratory ale less error prone comparing to novel technologies to be implemented. Also, while the NGS technologies for themselves are user friendly, the following bioinformatics analyses are too complex to be performed in a regular diagnostic laboratory. A solution would be to use experience of laboratories specialized in particular mutation detection. Still, relatively high cost of both NGS experiments and bioinformatics analyses may be a major limitation for large-scale application of NGS in diagnostic laboratory.

## Mosaicism and genetic counseling

Mosaicism can complicate clinical diagnosis and genetic counseling. Mosaic phenotypes may have incomplete syndromic features, which may stay unnoticed, especially in a low-grade mosaicism. The recurrence risk for unaffected parents who have an affected child and are considering a pregnancy may be influenced by the frequency of the mosaic mutation, the severity of the phenotype conferred by mosaicism, the type of mutational mechanism, or the sex and age of the mosaic parent (Lupski 2013; Campbell et al. 2014b).

Mutation load in the material tested does not necessarily correlate with the severity of disease as shown in the study by Donkervoort et al. (2015). A patient had the highest mutation load tested but showed the mildest phenotype, or vice versa the mosaic mutation was virtually undetectable in certain tissues (blood and saliva) in a clearly affected patient who tested positive in fibroblasts (Donkervoort et al. 2015). Choosing a single tissue only to be examined, there is always a possibility that higher levels of somatic mosaicism might be present in tissues derived from other cell lineages, but these were not analyzed, frequently due to unavailability of other tissues (Donkervoort et al. 2015).

Being currently revealed, low-grade somatic mosaicism appears to be a factor frequently causing diseases. Carriers of mosaic mutations may be at risk for abnormal pregnancy outcomes including offspring with the phenotype caused by the mutation in the form of a dominant allele. Those findings have important implications for genetic counseling and for understanding patterns of recurrence in transmission genetics. When two or more affected children are born to apparently unaffected parents, germline mosaicism is suspected. Identified mutation in the offspring suggests that dominant mutation appeared as recessive inheritance. However, with growing evidence of low-grade somatic mosaicism, without evaluation in somatic cells of the parents and/or the germ cells the diagnosis will be uncompleted. Consequently, regardless of mosaicism type, whether the parent has germline or somatic mosaic, he or she is at risk for a recurrence of another child with the disease. The risk ratios remain to be elucidated, although integrated probabilistic modeling of gametogenesis allowed predicting that mutations in parental blood increase recurrence risk substantially more than parental mutations confined to the germline (Campbell et al. 2014b).

Startling are the results in the newest report about low-level constitutional mosaicism identified in a patient with breast cancer. Identified low-level constitutional somatic mosaicism in the *BRCA1* (mutation c.1953dupG) indicated that the mutations recognized apparently in tumors only might be in fact present in low frequency in all body tissues as a constitutional genomic feature. As Sanger sequencing is the most frequently used method to screen *BRCA1* gene in patients and individuals in risk of cancer, this technique would miss the majority of *BRCA1* low rate mosaic mutations. Then, deep sequencing seems to be an optimal technique to screen this gene. Also, based on this example, it appears that constitutional mosaicism, similarly to constitutional heterozygous mutation in the carriers, should be screened and the carriers should be counseled for contralateral breast cancer, ovarian cancer and cancer risks for their offspring (Friedman et al. 2015).

As already mentioned, both low-grade somatic mosaicism and low-grade constitutional somatic mosaicism may occur early in embryonic development. Both low-grade somatic mosaicism and low-grade constitutional somatic mosaicism are comparable in one important diagnostic aspect: both types may be undetectable using DNA from PBS by Sanger sequencing. As different tissues should be tested to distinguish between the types, multiple samples are necessary to be proceeded. Although NGS technologies allow for low-grade mosaicism detection, a number of material samples to be tested per one patient are limited because of the high cost. Compared to targeted NGS, targeted Sanger sequencing is a low cost technique even if several samples from different body sites from one patient would be examined. Therefore, the solution would be to modify protocols including as an obligatory additional material in the form of buccal swabs, saliva, and/or nails for DNA extraction, and then fibroblast and other tissues, if necessary to verify the results.

The need to revise protocols of clinical diagnostics in various diseases has been already expressed in the diagnostic field as shown in examples of mosaicism investigation discussed in this paper in which an issue of appropriate biological material to be tested was highlighted. Although blood leukocytes have long been considered the “gold standard” sample for genetic testing, there were significant benefits to examine additionally other tissues. Study by Braunholz et al. revealed the high proportion of a mosaic *NIPBL* mutation in patients with a typical CdLS phenotype that were mutation negative in previous conventional Sanger sequencing approaches of DNA from blood or even BM tissue (Braunholz et al. 2015). The authors recommended adding BM as tested material and highlighted the need for use of highly sensitive technologies in molecular diagnostic of CdLS to improve genetic diagnosis and counseling of patients and their families (Braunholz et al. 2015). Also in the report of somatic mosaicism for dominant collagen 6 mutations, it was suggested that parental mosaicism may be more common than previously suspected in COL6-RD (Donkervoort et al. 2015). The authors concluded that caution is required, as low levels of somatic mosaicism may not be detectable by standard genetic sequencing and pure germline mosaicism will not be detectable by testing of specimen types routinely available to diagnostic laboratories (Donkervoort et al. 2015).

## Future directions

As NGS technologies constitute a promising methodological solution in mosaicism detection in the coming years, revisions in current diagnostic protocols are necessary to increase the detection rate of the unrevealed mosaicism events. In the NGS era, the apparently de novo mutations will be identified, in some percentage of cases, as a consequence of mosaicism occurred in the previous generation of the proband. Also, with various tissue samples tested per one individual mosaics even with low level of the mutation will be detected and the findings will allow for better genotype–phenotype correlations and more precise clinical diagnosis.
